# Spatial, environmental, and individual associations with *Anopheles albimanus* salivary antigen IgG in Haitian children

**DOI:** 10.3389/fcimb.2022.1033917

**Published:** 2022-11-08

**Authors:** Alicia Jaramillo-Underwood, Camelia Herman, Daniel Impoinvil, Alice Sutcliff, Alaine Knipes, Caitlin M. Worrell, LeAnne M. Fox, Luccene Desir, Carl Fayette, Alain Javel, Franck Monestime, Kimberly E. Mace, Michelle A. Chang, Jean F. Lemoine, Kimberly Won, Venkatachalam Udhayakumar, Eric Rogier

**Affiliations:** ^1^ Division of Parasitic Diseases and Malaria, Centers for Disease Control and Prevention, Atlanta, GA, United States; ^2^ Oak Ridge Institute for Science and Education (ORISE), Oak Ridge, TN, United States; ^3^ The Carter Center, Atlanta, GA, United States; ^4^ IMA World Health, Port-au-Prince, Haiti; ^5^ Programme National de Contrôle de la Malaria, Ministère de la Santé Publique et de la Population (MSPP), Port-au-Prince, Haiti

**Keywords:** *Anopheles albimanus*, multiplex serology, mosquito saliva, immunoglobulin G, *Plasmodium falciparum*

## Abstract

IgG serology can be utilized to estimate exposure to Anopheline malaria vectors and the Plasmodium species they transmit. A multiplex bead-based assay simultaneously detected IgG to *Anopheles albimanus* salivary gland extract (SGE) and four *Plasmodium falciparum* antigens (CSP, LSA-1, PfAMA1, and PfMSP1) in 11,541 children enrolled at 350 schools across Haiti in 2016. Logistic regression estimated odds of an above-median anti-SGE IgG response adjusting for individual- and environmental-level covariates. Spatial analysis detected statistically significant clusters of schools with students having high anti-SGE IgG levels, and spatial interpolation estimated anti-SGE IgG levels in unsampled locations. Boys had 11% (95% CI: 0.81, 0.98) lower odds of high anti-SGE IgG compared to girls, and children seropositive for PfMSP1 had 53% (95% CI: 1.17, 2.00) higher odds compared to PfMSP1 seronegatives. Compared to the lowest elevation, quartiles 2-4 of higher elevation were associated with successively lower odds (0.81, 0.43, and 0.34, respectively) of high anti-SGE IgG. Seven significant clusters of schools were detected in Haiti, while spatially interpolated results provided a comprehensive picture of anti-SGE IgG levels in the study area. Exposure to malaria vectors by IgG serology with SGE is a proxy to approximate vector biting in children and identify risk factors for vector exposure.

## Introduction

Malaria is an infectious disease caused by *Plasmodium* parasite infection in humans and is transmitted by mosquitoes in the genus *Anopheles.* In 2020, there were an estimated 241 million malaria infections globally, 0.26% of which were estimated to result in patient death (World Health Organization, 2021). While a disproportionate amount of disease burden lies in African countries, some areas of the world have made progress in reducing malaria transmission to the point of near elimination. Hispaniola, an island composed of the Dominican Republic and Haiti, is the only area in the Caribbean with endemic malaria, with *Plasmodium falciparum* as the primary species. Though recent malaria transmission in Haiti has been relatively low (Lucchi et al., 2014; Jules et al., 2022), heterogeneity by spatial, individual, and environmental factors accentuates the need for enhanced surveillance methods to characterize higher-risk regions and population subgroups to further move towards malaria elimination (Boncy et al., 2015; Cameron et al., 2021).

Reducing vectorial capacity is critical for any malaria elimination efforts (Briët et al., 2019). As the primary malaria vector in Haiti (Desenfant et al., 1987), *Anopheles albimanus* exhibits exophilic and exophagic preferences (Hobbs et al., 1986; Zimmerman, 1992; Frederick et al., 2016); peaks in vector density in Haiti occur after the start of the rainy seasons in November and June (Hobbs et al., 1986). Detailed knowledge of human-vector interaction in Haiti is limited, and only one published study to date has attempted to measure malaria transmission intensity using entomological inoculation rate, with no mosquitoes incriminated for *P. falciparum* (Hobbs et al., 1986). Traditional entomological methods may be inadequate in Haiti and other malaria low-transmission settings, pointing to the need for alternative methods that can provide better insights about vector biting in human populations.

Serologic methods have been used to assay for human antibody responses against antigens from both malaria vector and parasite. The multiplex bead-based assay (MBA) detects multiple analytes from the same specimen simultaneously (Elshal & McCoy, 2006) and has been utilized to detect antibodies against panels of *Plasmodium* antigens as evidence of an individual’s history of malaria infection (Kerkhof et al., 2015; Rogier et al., 2017; Rogier et al., 2019). Malaria vector exposure has also been elucidated using serologic methods *via* detection of antibody responses to Anopheline-specific antigens, which in turn provide information about vector contact among both individuals and populations (Bousema et al., 2010; Marie et al., 2020). In particular, immunoglobulin (Ig)G response to *Anopheles* salivary antigens has been used as a biomarker of exposure to mosquito bites, including in the areas where *An. albimanus* circulates (Londono-Renteria et al., 2010; Montiel et al., 2020), and has been compared against other measures of malaria transmission (Kearney et al., 2021). This current study utilized cross-sectional data collected in 2016 from a transmission assessment survey (TAS) conducted in Haiti with enrollment of children ages 6 and 7 at their schools (Druetz et al., 2020), and assessed risk factors and spatial associations for IgG against *An. albimanus* salivary antigens.

## Materials and methods

### Human subjects

Children were enrolled and samples were collected in 2016 as part of lymphatic filariasis (LF) transmission assessment surveys in Haiti, with integration of malaria RDTs and microscopy for soil-transmitted helminths in stool specimens (Knipes et al., 2017). Individuals with a positive RDT result received free treatment as per the national policy in Haiti. The study protocol was approved by the National Bioethics Committee of Haiti, and this activity was considered a program evaluation activity by CDC Human Subjects Office (#2014–256). Before enrollment, informed consent forms were sent home with each of the students so that a parent or legal guardian could read and sign for return to the study teams. Before sample collection, the informed consent forms were signed by the child’s parent, and verbal assent was given by the child for collection of multiplex serological data.

### Participant enrollment

Surveys were conducted in evaluation units (EUs) that had met World Health Organization (WHO) criteria to conduct an LF TAS, with the current WHO recommendation to conduct a school-based TAS in areas where the net primary-school enrollment rate is ≥75%. Haitian school enrollment data for 2014 were utilized along with population census data to determine the sampling approach employed in each EU, which are program defined and dependent on baseline LF prevalence found during initial mapping surveys (Beau de Rochars et al., 2004). Per WHO criteria, only children aged 6 or 7 years were approached to participate, and after verbal assent, fingerpick blood was collected on filter papers (TropBio filter wheels, Cellabs, Sydney, Australia), dried (creating a dried blood spot, DBS), and packaged individually with desiccant for later laboratory analysis at the U.S. Centers for Disease Control and Prevention in Atlanta, GA.

### Mosquito dissection, SGE preparation, and bead conjugation

As described previously (Jaramillo-Underwood et al., 2022), whole salivary gland pairs were dissected from three- to seven-day-old unfed *An. albimanus* mosquitoes (lab strain STECLA) and frozen for later use. Whole salivary glands were homogenized with glass tissue grinder in phosphate buffered saline (PBS, pH 7.2) and freeze-thawed twice for further protein dissociation. Total protein concentration of this SGE homogenate was determined by BCA assay (Pierce BCA Protein Assay Kit, ThermoFisher).

Six separate bead regions (Bio-Plex non-magnetic beads, BioRad, Hercules, CA) were coupled with antigens for IgG capture and subsequent detection. Recombinant *Schistosoma japonicum* glutathione-*S*-transferase (GST) was utilized as a non-binding internal well control and was coupled at pH 5.0 at 20 μg/mL. The malaria antigens in the multiplex panel have all been reported before: the *P. falciparum* merozoite surface protein 1 19kD fragment (PfMSP1_19_; coupled at pH 5.0 at 20 μg/mL), *P. falciparum* apical membrane antigen 1 N-terminal region (PfAMA1; coupled at pH 5.0 at 20 μg/mL), *P. falciparum* circumsporozoite protein (NANP)_5_ peptide fused to GST (CSP; coupled at 30 μg/mL), and Pl1043 epitope from *P. falciparum* liver stage antigen 1 (LSA-1) (Rogier et al., 2015; Plucinski et al., 2018). The homogenate of salivary gland proteins was conjugated to beads as described previously at pH 5.0 at 30 μg/mL.

### Assay for anti-SGE and *P. falciparum* IgG by multiplex bead assay

Participant whole blood was eluted from a single tab of the TropBio filter wheels to provide sample for the IgG detection assay. A single DBS tab (10 μL whole blood) was rehydrated in blocking buffer (PBS pH 7.2, 0.5% Polyvinyl alcohol (SigmaAldrich, St. Louis, MO), 0.5% polyvinylpyrrolidine (SigmaAldrich), 0.1% casein (ThermoFisher, Waltham, MA), 0.5% bovine serum albumin (SigmaAldrich), 0.3% Tween 20, 0.05% sodium azide, and 0.01% *E*. *coli* extract to prevent non-specific binding) and diluted to a final concentration of 1:200, which is approximately a 1:400 serum dilution. Diluted blood samples were stored at 4°C until assayed.

All IgG assay reagents were diluted in buffer containing PBS, 0.05% Tween 20, 0.5% bovine serum albumin (SigmaAldrich), and 0.02% NaN_3_. Positive and negative controls, which had been determined by preliminary assay runs as high or low responders, were included on each IgG detection plate to ensure appropriate assay performance. The bead mix including all bead regions contained approximately 1,000 beads/region added to each assay well. Samples (50 μL of 1:200 dilution of eluted whole blood) were incubated with beads for 90 min at room temperature under gentle shaking protected from light in MultiScreen-BV filter plates (SigmaAldrich). After three washes (wash buffer: PBS, 0.05% Tween 20) following incubation, beads were incubated with 50 μL biotinylated detection antibody (a mixture of 1:500 anti-hIgG and 1:625 anti-hIgG_4_, both produced by Southern Biotech, Birmingham, AL) for 45 min. After three washes, 50 μL streptavidin-phycoerythrin (Invitrogen, Waltham, MA) were added to all wells (1:250x of 1 mg/mL) for a 30-min incubation. After three washes, samples beads were incubated with 50 μL reagent buffer for 30 min, washed once, and resuspended in 100 μL PBS. Assay plates were briefly shaken and read on a Bio-Plex 200 machine (BioRad) by generating the median fluorescence intensity (MFI) for a target of 50 beads. The final measure, denoted as MFI minus background (MFI-bg), was reported by subtracting MFI values of beads on each plate only exposed to sample diluent during the first incubation step. The MFI-bg threshold for true positive IgG assay signal against *Plasmodium* antigens was ascertained if the sample MFI-bg was higher than the mean + 3SD of the MFI-bg signal of a panel of 92 known anti-malaria IgG negative DBS samples.

### Statistical analysis

Statistical analysis was performed using SAS (version 9.4; SAS Institute Inc., Cary, USA). Figures were produced using R Statistical Software (version 4.1.1; R Foundation for Statistical Computing, Vienna, Austria) and GraphPad Prism (version 9.3.1; GraphPad Software, San Diego, USA). Individual covariates included sex; rapid diagnostic test (RDT, First Response Malaria Histidine-Rich Protein II (HRP2); II3FRC30, Premier Medical Corporation, New Jersey) result; and seropositivity to four *P. falciparum* antigens, PfAMA1, CSP, LSA-1, and PfMSP1. Environmental covariates by school GPS coordinate included elevation, normalized difference vegetation index (NDVI), population density, rainfall, distance to the nearest water body (defined as the nearest stream, river, or lake), and air temperature (**Figure S1**). Data for environmental covariates were obtained from outside sources and values corresponding to each school’s GPS coordinates were sampled using QGIS (v3.20.3-Odense); any values with a temporal component were averaged across the study period (**Table S1**). Log-transformed MFI-bg values represent anti-SGE IgG levels as a proxy for vector exposure. Student’s t-test assessed differences in mean vector exposure by individual covariates. Non-parametric Mann-Whitney U and Kruskal-Wallis tests assessed categorical differences by environmental covariates using an empirical Bayes estimate of mean vector exposure by school; Spearman’s correlation coefficient assessed the relationship between vector exposure and continuous values of environmental covariates. A multilevel logistic regression model provided estimates of prevalence odds ratios, using an outcome of an above-median anti-SGE IgG response that adjusted for covariates at the individual and environmental level.

### Spatial analysis

Spatial analysis was conducted in SaTScan v10.0.2 to detect statistically significant clusters of schools with students who had elevated anti-SGE IgG responses using a Bernoulli probability model. Due to a lack of sampling in all regions of the country, clusters were detected separately in northern (Nord and Artibonite departments as well as La Tortue, which is in Nord-Ouest department) and southern (Grand’Anse and Sud departments) regions of Haiti. Outputs were overlayed onto geospatial surfaces of Haiti using QGIS. Spatial interpolation of anti-SGE IgG levels to unsampled locations within the TAS study area was conducted in QGIS using inverse distance weighting; select environmental variables were depicted for visual comparison with the spatially interpolated results.

## Results

### Study population

From the 2016 Haiti TAS, 11,541 students had samples available for the multiplex IgG assay, representing 350 schools of enrollment (**Figure S2**). Of students enrolled who had sex and age data available, 51.8% of participants were female and 55.9% were 7 years old (**Table 1**). At the time of enrollment, 16 (0.2%) children were positive for *P. falciparum* malaria infection by RDT, while 919 (8.0%) had a positive IgG response against any *P. falciparum* antigen. Overall, seropositivity to PfAMA1 (3.8%) and PfMSP1 (4.9%) was more common than seropositivity to CSP (0.8%) and LSA-1 (0.4%). Compared to other departments, Grand’Anse had the highest proportion of participants who were RDT positive (13/810 RDT positive, 1.6%) and had past malaria exposure (328/1650 P*. falciparum* IgG positive, 19.9%). The median elevation of schools was 208 m (IQR: 62-346 m); the median distance to the nearest water body was 2.2 km (IQR: 0.8-4.5 km); and the median normalized difference vegetation index (NDVI) was 0.6 (IQR: 0.5-0.7), indicating relatively dense vegetation throughout the study area (**Table S2**) (Brown, 2018).

**Table 1:** Descriptive statistics of the study population: Haiti, 2016.

	Overall	Artibonite	Grand’Anse	La Tortue*	Nord	Sud
**Total**	**11541**	**3153**	**1650**	**848**	**3656**	**2234**
Female	5739 (51.8)	1627 (51.8)	787 (49.2)	419 (49.8)	1736 (52.2)	1170 (53.7)
Age						
6 years	4888 (44.1)	1253 (39.9)	688 (43.0)	344 (40.9)	1479 (44.5)	1124 (51.5)
7 years	6199 (55.9)	1887 (60.1)	912 (57.0)	498 (59.1)	1845 (55.5)	1057 (48.5)
Positive RDT^†^	16 (0.2)	0 (0.0)	13 (1.6)	0 (0.0)	0 (0.0)	3 (0.2)
CSP seropositive	86 (0.8)	14 (0.4)	23 (1.4)	2 (0.2)	23 (0.6)	24 (1.1)
LSA-1 seropositive	50 (0.4)	3 (0.1)	33 (2.0)	1 (0.1)	2 (0.1)	11 (0.5)
PfAMA1 seropositive	441 (3.8)	83 (2.6)	168 (10.2)	21 (2.5)	85 (2.3)	84 (3.8)
PfMSP1 seropositive	564 (4.9)	81 (2.6)	248 (15.0)	6 (0.7)	106 (2.9)	123 (5.5)
Seropositive to any *P. falciparum* antigen	919 (8.0)	167 (5.3)	328 (19.9)	29 (3.4)	189 (5.2)	206 (9.2)

Data presented as n (%).

*La Tortue is the only commune in Nord-Ouest department for which data were collected.

^†^Rapid diagnostic test.

The bold values indicate cumulative total numbers for each column.

### Individual covariates

**Figure 1:**
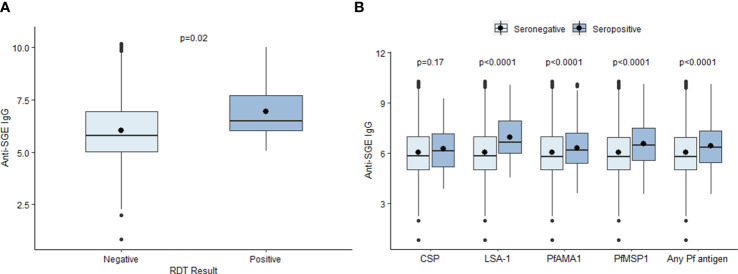
Relationship between select individual factors and anti-SGE IgG levels. **(A)** Boxplots of log-transformed salivary gland extract (SGE) immunoglobulin (Ig)G levels by malaria infection at time of enrollment, indicated by rapid diagnostic test (RDT) result. **(B)** Boxplots of anti-SGE IgG levels by seropositivity to *P. falciparum* antigen targets. Boxes represent the interquartile range (IQR) of anti-SGE IgG values for each category; the horizontal line in each box is the median anti-SGE IgG value and the circle represents the mean anti-SGE IgG. Whiskers extend 1.5x IQR above and below boxes, and circles represent outlier anti-SGE IgG values outside of 1.5x IQR.

The mean log-transformed MFI-bg value for anti-SGE IgG was 6.07 (SD = 1.36) in the study population. The mean anti-SGE IgG level was significantly higher among six-year-olds (6.14, SD = 1.40) compared to seven-year-olds (6.00, SD = 1.33) (t = 5.37, p<0.0001) (**Figure S3A**), and there was no significant difference in anti-SGE IgG levels based on sex (t = -1.47, p = 0.14) (**Figure S3B**). Children positive for *P. falciparum* infection by RDT at time of enrollment had significantly higher anti-SGE IgG levels (6.92, SD = 1.38) compared to those without (6.01, SD = 1.34) (t = -2.64, p = 0.02) (**Figure 1A**). Children seropositive to LSA-1, PfAMA1, and PfMSP1 antigens had significantly higher anti-SGE IgG levels compared to those who were seronegative to each antigen (t = -4.66, p < 0.0001; t = -4.41, p < 0.0001; and t = -9.50, p < 0.0001, respectively), while there was no difference in anti-SGE IgG between children seropositive to the *P. falciparum* sporozoite protein CSP and those seronegative (t = -1.39, p = 0.17) (**Figure 1B**). Seropositivity to any of the four *P. falciparum* antigen targets was associated with significantly higher anti-SGE IgG levels (6.44, SD = 1.30) compared to those who were seronegative to all *P. falciparum* antigens (6.04, SD = 1.36) (t = -8.93, p<0.0001).

### Environmental covariates

**Figure 2:**
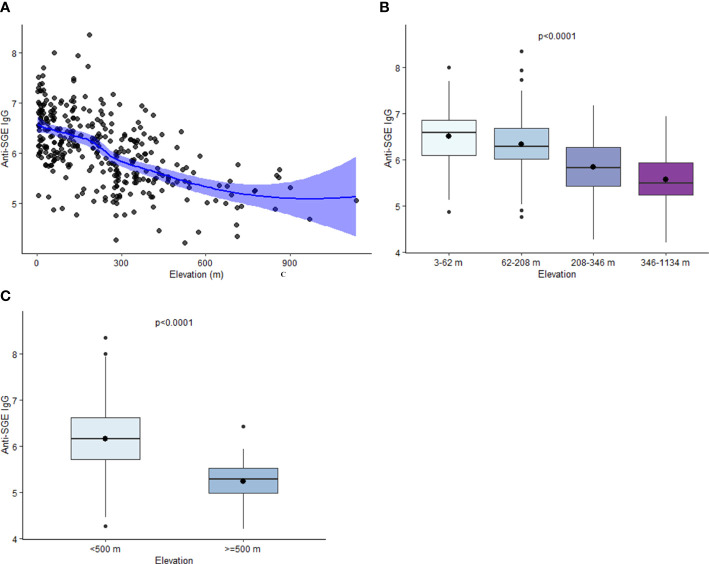
Relationship between elevation and anti-SGE IgG levels. **(A)** Loess curve of the relationship between log-transformed salivary gland extract (SGE) immunoglobulin (Ig)G and elevation. The line represents a fitted smooth curve between elevation and anti-SGE IgG. Shading depicts the range of values that contain the true range of anti-SGE IgG values by elevation with 95% confidence. **(B)** Boxplots of anti-SGE IgG levels by elevation (m) are grouped into quartiles. **(C)** Boxplots of anti-SGE IgG levels for all schools at elevations above and below 500 m. For plots **(B, C)**, boxes represent the interquartile range (IQR) of anti-SGE IgG values for elevation categories, the horizontal line represents the median anti-SGE IgG value, and the circle represents the mean anti-SGE IgG value. Whiskers extend 1.5x IQR above and below boxes, and circles represent outlier anti-SGE IgG values outside of 1.5x IQR.

Estimated mean values of anti-SGE IgG from children enrolled in different schools varied from 4.2 to 8.4. A negative relationship was observed between elevation and anti-SGE IgG by school (r_s_ = -0.57, p < 0.0001) (**Figure 2A**); when elevation was grouped into quartiles, IgG levels significantly decreased with increasing elevations (X^2^ = 101.1, p < 0.0001) (**Figure 2B**). In just comparing those who were enrolled at schools under 500 m versus those over 500 m in elevation, anti-SGE IgG was substantially higher at lower elevation (Z = -7.02, p < 0.0001) (**Figure 2C**). Negative correlations were observed between NDVI and anti-SGE IgG levels (r_s_ = -0.09, p = 0.09) and distance to the nearest body of water (r_s_ = -0.18, p = 0.0009). Rainfall (r_s_ = 0.23, p < 0.0001), population density (r_s_ = 0.32, p < 0.0001), and temperature (r_s_ = 0.35, p < 0.0001) were positively correlated with anti-SGE IgG.

### Adjusted model for associations with anti-SGE IgG

**Figure 3:**
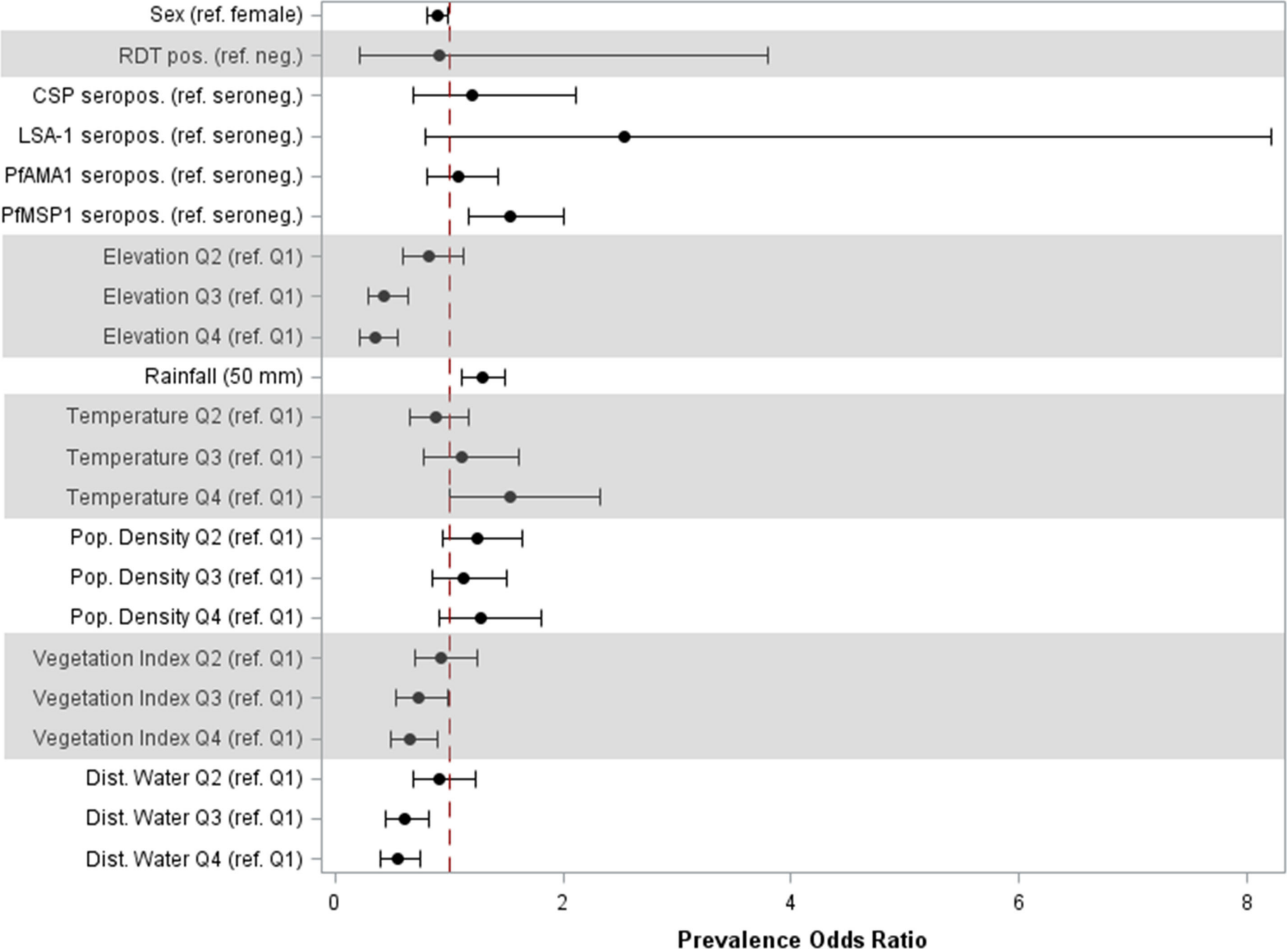
Prevalence odds ratio estimates for individual and environmental factors associated with high anti-SGE IgG levels. Point estimates and 95% confidence intervals are displayed for main effects of multilevel logistic regression. Individual-level effects include sex; rapid diagnostic test (RDT) result; and seropositivity to *P. falciparum* antigens CSP, LSA-1, PfAMA1, and PfMSP1. Environmental-level effects include elevation, rainfall, temperature, population density, normalized difference vegetation index, and distance to the nearest water body; variables that did not have a linear relationship with the logit were categorized into quartiles.

In the adjusted model, sex and seropositivity to PfMSP1 were the only individual factors found to be significantly associated with high anti-SGE IgG levels (**Figure 3**, **Table S3**). Compared to girls, boys had 11% (95% CI: 0.81, 0.98) lower odds of high IgG levels, and children seropositive to PfMSP1 had 53% (95% CI: 1.17, 2.00) higher odds of elevated anti-SGE IgG compared to those who were PfMSP1 seronegative. Among environmental covariates, with the referent as the lowest elevation quartile, quartiles 2, 3, and 4 of increasing elevation had successively lower odds [aPOR: 0.81 (95% CI: 0.58, 1.12), 0.43 (95% CI: 0.29, 0.63), and 0.34 (95% CI: 0.22, 0.54), respectively] of elevated anti-SGE IgG. The same relationship was observed with NDVI and distance to the nearest water body when grouped into quartiles, where higher quartiles for both covariates were associated with lower odds of having high anti-SGE IgG when compared to the lowest quartile. Increased rainfall was positively associated with higher anti-SGE IgG levels; for each additional 50 mm of average rainfall, odds increased by an estimated 29% (95% CI: 1.12, 1.49). Estimates for temperature indicated successively higher odds of high anti-SGE IgG at the top quartiles of temperature values [aPOR_Q3_: 1.11 (95% CI: 0.77, 1.61); aPOR_Q4_: 1.52 (95% CI: 1.00, 2.31)] compared to the lowest quartile.

### Spatial analysis

**Figure 4:**
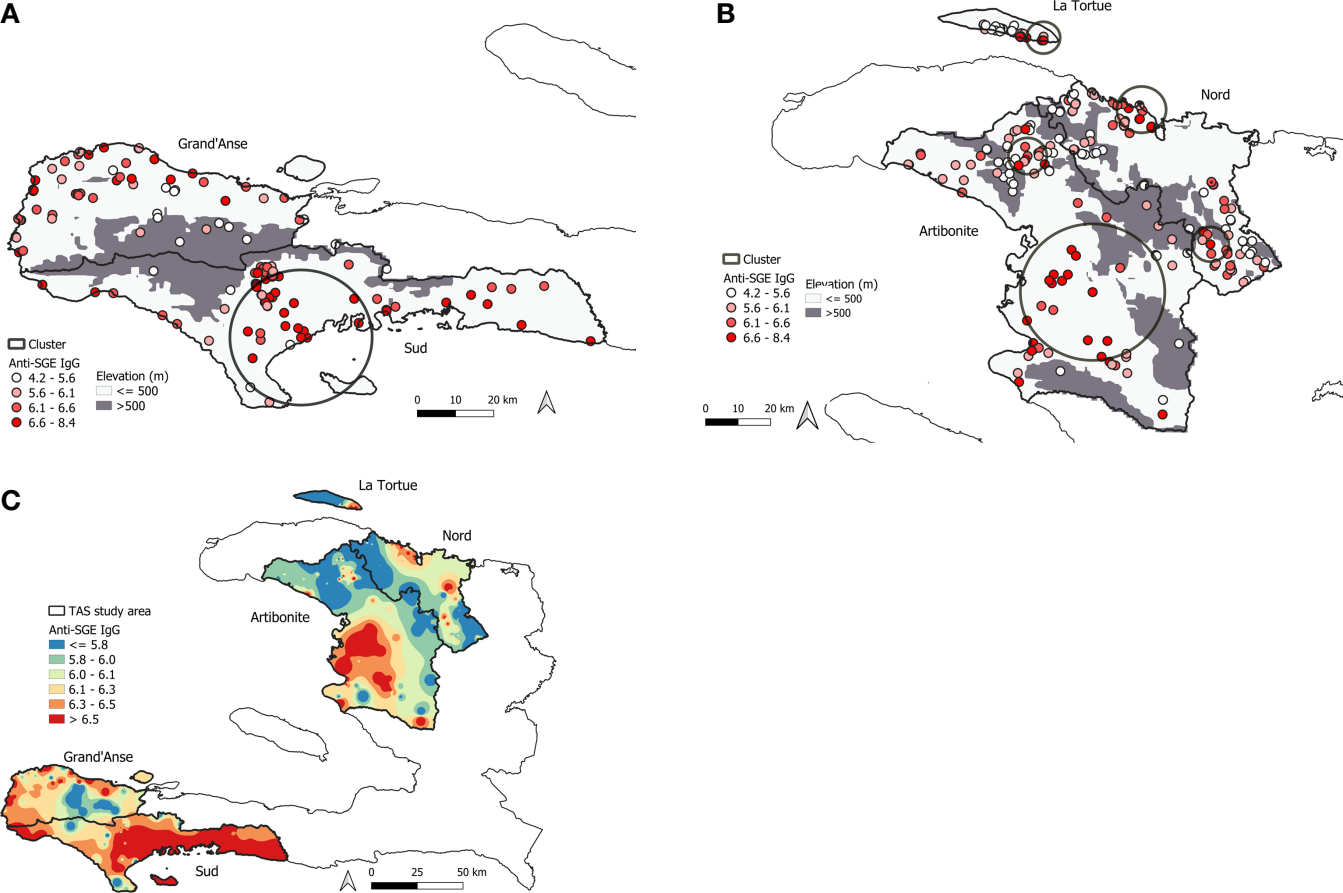
Mapping of high *An. albimanus* anti-SGE IgG levels in Haiti. **(A)** Significant spatial cluster of high anti-SGE IgG responses located in southern Haiti, encompassing 130 schools in Grand’Anse and Sud departments. **(B)** Significant spatial clusters of high anti-SGE IgG responses located in the northern region of Haiti, encompassing 220 schools in Artibonite and Nord departments and the island of La Tortue. Small, solid circles represent individual schools, with darker shading to indicate greater mean anti-SGE IgG levels by school. Elevations above 500 m are represented in grey to distinguish Anopheline preference. **(C)** Heat map of inverse-distance weighting interpolation of schools’ lognormal mean anti-SGE IgG levels within the TAS study area.

In total, seven statistically significant clusters were identified in the study area with children who had elevated anti-SGE IgG levels (**Table S4**). One cluster was detected in the southern peninsula (**Figure 4A**), while six were detected in northern Haiti (**Figure 4B**), with one cluster in Nord department with a radius of 0.1 km not visible on the map. Spatially interpolated intensity of anti-SGE IgG levels (**Figure 4C**) showed that higher levels of anti-SGE IgG generally corresponded to areas of lower elevation and coastlines. Visual representation of elevation, average NDVI, and average rainfall levels in Haiti are shown in **Figure S4**.

## Discussion

Malaria infection occurs through injection of *Plasmodium* sporozoites within mosquito saliva during a blood meal, and estimating exposure to malaria vectors represents an indirect assessment of malaria risk where *Plasmodium* parasites are endemic (Vanderberg and Frevert, 2004). Detecting IgG against salivary proteins to estimate bite exposure has been described as a potential complement to traditional methods, such as the entomological inoculation rate and human biting rate (Londono-Renteria et al., 2010; Kearney et al., 2021). However, few studies have characterized human antibody response to *Anopheles* salivary antigens in large sample sizes, and most of these studies have focused on the *An. gambiae* salivary gland 6 (gSG6) antigen (Poinsignon et al., 2008; Londono-Renteria et al., 2010; Drame et al., 2015; Ya-Umphan et al., 2018; Montiel et al., 2020). Results for different salivary antigens, immunoassay platforms, and study populations have varied regarding age, with some finding that IgG increases with age (Drame et al., 2010; Drame et al., 2013), decreases with age (Londono-Renteria et al., 2010; Rizzo et al., 2011), or that there is no association between IgG against salivary proteins and age (Waitayakul et al., 2006; Idris et al., 2017). Our group previously reported on the capacity of SGE homogenate from the *An. albimanus* STECLA strain to capture human IgG, with stark contrasts in IgG levels by age with peak in IgG levels at ages 6 and 7 (Jaramillo-Underwood et al., 2022). This current study’s enrollment of a large number of children ages 6 and 7 and inclusion of other individual and environmental factors provides a clear evaluation of how IgG against salivary proteins can be used to predict Anopheline exposure and assess risk of exposure to malaria vectors in low-transmission settings.

While there was no difference in level of anti-SGE IgG by sex in crude analysis, adjusted results indicated that boys had 11% significantly lower odds of prevalence of high anti-SGE IgG compared to girls. In contrast, a study conducted in Artibonite, Haiti, found that males typically had elevated anti-SGE IgG levels compared to females, though this finding applied to all ages and was not statistically significant (Jaramillo-Underwood et al., 2022). Other groups have similarly found that males were bitten more frequently by Anophelines than females, which was related to the greater amount of time they spent outdoors compared to females (Camargo et al., 1996; Pathak et al., 2012; Guglielmo et al., 2021). Indeed, while *An. albimanus* has been shown to exhibit indoor biting behavior (Bown et al., 1993), it is widely considered to be exophilic (Hobbs et al., 1986; Zimmerman, 1992; Ryan et al., 2017). Further investigation of this age- and sex-based association is needed, including adjustments for outdoor activity levels and other potential confounders.

Though findings from this study point to a significant association only between IgG against PfMSP1 and anti-SGE IgG in the adjusted model, seropositivity to each of the four *P. falciparum* antigens was consistently associated with increased IgG against salivary proteins in bivariate analyses. This is in line with previous findings from the Thailand-Myanmar border, which found significant, positive associations between PfMSP1 and CSP seroprevalence and seroprevalence to gSG6-P1 (Ya-Umphan et al., 2018), but stands in contrast to other studies that have not found a significant association between anti-*Anopheles* IgG and seroprevalence to PfMSP1 (Londono-Renteria et al., 2020; Jaramillo-Underwood et al., 2022). The present study included *Plasmodium* blood-stage and pre-erythrocytic stage antigens, each having varying levels in humans and longevity of IgG antibodies in circulation (Ya-Umphan et al., 2018). Studies in murine models have found that IgG against Anopheline salivary antigens has modulatory effects on the host’s immune response to malaria infection (Cross et al., 1993; Schneider et al., 2011). While a study of children in Senegal found differential acquisition of IgG responses against *P. falciparum* antigens depending on intensity of *Anopheles* exposure (Sarr et al., 2012), it remains to be established what mechanism is responsible for this difference—and caution must be used when interpreting results restricted to children. Seropositivity to PfAMA1 and PfMSP1 could serve as a reliable proxy for lifetime malaria exposure among young children, while seropositivity to CSP and LSA-1 likely indicates malaria exposure within the past few months (Ondigo et al., 2014). Regardless of IgG dynamics to these different *P. falciparum* antigens in children in this study, higher IgG levels at a population level would indicate more people being exposed to *P. falciparum.* Further investigation is necessary to understand the associations between short- and long-term malaria exposure and vector exposure, especially among different age groups.

When adjusting for other variables, RDT result was not significantly associated with high anti-SGE IgG. While the number of RDT positive children (n=16) was insufficiently large to explore associations by infection status, it was nonetheless an expected finding in Haiti’s low-transmission setting. This points to the importance of using other markers of exposure to assess malaria risk when infection is rare; while 0.2% of the study population had a positive RDT, 8% of study participants—and 20% of participants in Grand’Anse department—were IgG positive to *P. falciparum* antigens, indicating the need for serologic methods in assessing both malaria and vector exposure in this type of setting.

These data link associations between vector exposure (through the anti-SGE IgG biomarker) and a variety of environmental factors. Anopheline mosquitoes prefer breeding sites below 500 m in elevation (Rubio-Palis and Zimmerman, 1997), and the strongly negative dose-response relationship found in both bivariate and adjusted analyses between enrollment site elevation and anti-*Anopheles* IgG supports these previous findings. Indeed, at lower elevations, there are more possibilities for standing water sites where these mosquitoes can complete their life cycles (Mattah et al., 2017). While data were unavailable for small aquatic habitats such as puddles and ditches, a negative dose-response association was still found between increased distance to larger water bodies and anti-SGE IgG. Higher vegetation levels were also associated with lower anti-SGE IgG, contrasting with previous findings indicating that increased vegetation is associated with enhanced mosquito survival (Stone et al., 2012). However, knowledge of the resting, host-seeking, and biting behavior of mosquitoes in Haiti is limited (Frederick et al., 2016), and studies of vegetation in other malaria settings may not apply to Haiti—leaving this result open to further exploration. Positive associations between anti-SGE IgG and rainfall and temperature, meanwhile, are in line with current understanding; while rainfall leads to development of suitable breeding sites, higher temperatures tend to occur at lower elevations and allow larvae maturation. Based on these strong associations, rainfall and elevation data alone may be sufficient for estimating vector exposure in Haiti as shown by antibody responses to SGE.

The regions of Haiti included as school sampling sites have high heterogeneity in malaria disease burden and vector density (Frederick et al., 2016; Cameron et al., 2021). High levels of vector exposure can still occur even when *P. falciparum* is not prevalent in the human population. Visual comparison between spatially interpolated results and a map of Haiti’s elevation shows that highest levels of anti-SGE IgG matched well to areas of low elevation across the TAS study area; this also corresponds with the strong relationships observed between elevation and anti-SGE IgG in both bivariate and multivariate analyses. Because the other environmental variables used in this study were temporally averaged, whereas elevation has static values, this may not only account for the weak correlations observed in bivariate analyses, but also for the lack of a clear relationship with anti-SGE IgG levels in spatial analysis. The low resolution of rainfall data relative to other environmental variables also makes comparison difficult with interpolated anti-SGE IgG. More complex geostatistical analyses could further evaluate these relationships.

This study was subject to multiple limitations. Because participant enrollment was restricted to participants ages 6-7, the main limitation is generalizability of our results to other age groups in Haiti or beyond. As IgG serological data is a proxy for vector exposure, another limitation to this study is the association between anti-SGE IgG and environmental covariates. Because enrollment was at schools, it is impossible to know the exact location where exposure to *An. albimanus* bites was occurring, as bites could have occurred anywhere outside the school where children enrolled. However, it is reasonable to assume that most children’s households were likely within a few kilometers of the school they were attending. Another limitation is the lack of data on entomological measures such as human biting rate (HBR) and entomological inoculation rate (EIR), which would have been useful for validating anti-SGE IgG as a biomarker for both vector exposure and malaria transmission; future directions would include collection of these data for analysis. Moreover, while the RDT was the most practical tool available to indicate active infection in this study, RDTs may not have captured all of the very-low-density *P. falciparum* infections in this non-treatment-seeking population. It is thus possible that a more-sensitive diagnostic tool such as polymerase chain reaction (PCR) could have detected more infections in this setting, allowing for a more robust analysis by infection status (Wu et al., 2015). Additionally, mosquito salivary gland extracts are crude and therefore reactivity is likely to multiple factors, including unknown immunogenic factors. The data generated here are specific for the extract used in the study only and there are no guarantees that other extracts will produce the same reactivities. Finally, the cross-sectional study design did not allow for examining variations in vector exposure across time and seasonal differences, including characterization by wet and dry seasons.

The current study presents results on associations with vector exposure among school-aged children, as estimated by IgG levels against Anopheline salivary proteins. Findings indicate that environmental, serologic, and demographic variables alike have varying degrees of association with vector exposure in this group, while spatial analysis can be used to estimate exposure in unsampled areas. Taken together, these results point to more-targeted capabilities for predicting and addressing malaria risk in low-transmission settings using serological indicators of vector exposure along with other traditional parameters.

## Data availability statement

The raw data supporting the conclusions of this article will be made available by the authors, without undue reservation.

## Ethics statement

The studies involving human participants were reviewed and approved by National Bioethics Committee of Haiti. Written informed consent to participate in this study was provided by the participants’ legal guardian/next of kin.

## Author contributions

AK, CW, LF, LD, CF, AJ, FM, JL, and KW coordinated field studies. CH and ER collected laboratory data. DI and AS provided mosquito salivary glands. VU and KW provided laboratory support. KM and MC provided malaria expertise. AJ-U performed formal analysis. AJ-U and ER drafted manuscript. All authors contributed to the article and approved the submitted version.

## Acknowledgments

We acknowledge the communities in Haiti who enabled this work to take place. The authors are grateful to BEI Resources for providing the *Anopheles albimanus* STECLA mosquitoes, MRA-126, contributed by Mark Benedict.

## Conflict of interest

The authors declare that the research was conducted in the absence of any commercial or financial relationships that could be construed as a potential conflict of interest.

## Publisher’s note

All claims expressed in this article are solely those of the authors and do not necessarily represent those of their affiliated organizations, or those of the publisher, the editors and the reviewers. Any product that may be evaluated in this article, or claim that may be made by its manufacturer, is not guaranteed or endorsed by the publisher.

## Author disclaimer

The findings and conclusions in this report are those of the authors and do not necessarily represent the official position of the Centers for Disease Control and Prevention (CDC).
