# Supplementary material for: Spatial, environmental, and individual associations with Anopheles albimanus salivary antigen IgG in Haitian children
Source: Front Cell Infect Microbiol. 2022 Nov 8;12:1033917. doi: 10.3389/fcimb.2022.1033917 (PMC9681116; doi:10.3389/fcimb.2022.1033917)
Supplement: Supplementary file 1 [file DataSheet_1.docx]

Supplementary Material

# Supplementary Tables

**Table S1.** Remote sensing data: resolutions, units, and sources

| **Predictor** | **Unit** | **Spatial Resolution** | **Temporal Resolution** | **Source** |
| --- | --- | --- | --- | --- |
| Air temperature | °C | 0.05 decimal degrees | 1 month | Accessible by figshare* |
| Distance to nearest water body | m | N/A | N/A | Digital Chart of the World^†^ |
| Elevation | m | 90 m | N/A | CGIAR SRTM^‡^ |
| Normalized difference vegetation index | ratio | 250 m | 1 dekad | USGS^§^ |
| Population density | population / km^2^ | 1 km^2^ | 1 year | WorldPop^¶^ |
| Rainfall | mm | 0.05 x 0.05 degree | 1 dekad | CHIRPS^#^ |

*https://doi.org/10.6084/m9.figshare.c.4081802.v1

^†^https://docs.generic-mapping-tools.org/6.1/datasets/dcw.html

^‡^https://bigdata.cgiar.org/srtm-90m-digital-elevation-database/

^§^https://earlywarning.usgs.gov/fews/product/447

^¶^https://www.worldpop.org/geodata/listing?id=77

^#^https://data.chc.ucsb.edu/products/CHIRPS-2.0/global_monthly/tifs/

**Table S2.** Descriptive statistics at the environmental level of the study population: Haiti, 2016.

|  | **Overall** | **Artibonite** | **Grand’Anse** | **La Tortue*** | **Nord** | **Sud** |
| --- | --- | --- | --- | --- | --- | --- |
| **Number schools** | **350** | **96** | **55** | **26** | **98** | **75** |
| Distance to nearest water body (km) | 2.2 [0.8-4.5] | 1.2 [0.5-2.6] | 4.4 [1.8-8.7] | 12.7 [9.8-14.2] | 2.8 [0.9-4.3] | 1.6 [0.5-2.9] |
| Elevation (m) | 208 [62-346] | 240 [77-416] | 108 [63-315] | 241 [110-283] | 284 [59-373] | 165 [42-234] |
| Normalized difference vegetation index^†^ | 0.6 [0.5-0.7] | 0.5 [0.4-0.5] | 0.7 [0.6-0.8] | 0.8 [0.7-0.8] | 0.7 [0.5-0.8] | 0.7 [0.6-0.7] |
| Population density (per km^2^) | 366 [219-745] | 331 [215-722] | 268 [174-755] | 227 [176-364] | 423 [293-929] | 410 [255-790] |
| Rainfall^†^ (mm) | 104 [79-145] | 81 [59-104] | 149 [125-159] | 44 [41-44] | 107 [92-138] | 179 [108-201] |
| Temperature^†^ (°C) | 25.8 [25.3-26.3] | 25.8 [25.4-26.2] | 26.0 [25.7-26.5] | 26.4 [26.4-26.9] | 25.4 [25.3-25.8] | 26.0 [25.6-26.3] |

Data presented as median [IQR].

*La Tortue is the only commune in Nord-Ouest department for which data were collected.

^†^Average for February-June and August 2016

**Table S3.** Parameter estimates of prevalence odds ratios for main effects of multilevel logistic regression*

| **Effect** | **Estimate** | **aPOR** | **95% CI** | | ***p*-value** | |
| --- | --- | --- | --- | --- | --- | --- |
|  |  |  | **Lower** | **Upper** |  | |
| **Sex (ref. female)** | **-0.11** | **0.89** | **0.81** | **0.98** | **0.02** | |
| Positive RDT^†^ (ref. negative) | -0.09 | 0.92 | 0.22 | 3.80 | 0.90 | |
| ***P. falciparum* seropositivity**  **(ref. seronegative)** |  |  |  |  |  | |
| CSP | 0.18 | 1.20 | 0.68 | 2.11 | 0.53 | |
| LSA-1 | 0.94 | 2.55 | 0.79 | 8.24 | 0.12 | |
| PfAMA1 | 0.08 | 1.08 | 0.81 | 1.43 | 0.60 | |
| **PfMSP1** | **0.43** | **1.53** | **1.17** | **2.00** | **0.002** | |
| **Elevation (m) (ref. Q1)** |  |  |  |  |  | |
| Q2 | -0.21 | 0.81 | 0.58 | 1.12 | 0.21 | |
| **Q3** | **-0.85** | **0.43** | **0.29** | **0.63** | **<0.0001** | |
| **Q4** | **-1.07** | **0.34** | **0.22** | **0.54** | **<0.0001** | |
| **Distance (m) to nearest water body (ref. Q1)** |  |  |  |  |  | |
| Q2 | -0.12 | 0.89 | 0.66 | 1.20 | 0.45 | |
| **Q3** | **-0.51** | **0.60** | **0.44** | **0.81** | **0.001** | |
| **Q4** | **-0.61** | **0.54** | **0.40** | **0.74** | **0.0001** | |
| **Normalized difference vegetation index (ref. Q1)** |  |  |  |  |  | |
| Q2 | -0.06 | 0.94 | 0.70 | 1.26 | 0.67 | |
| **Q3** | **-0.31** | **0.73** | **0.54** | **0.99** | **0.05** | |
| **Q4** | **-0.41** | **0.66** | **0.49** | **0.90** | **0.008** | |
| Population density (per km^2^) (ref. Q1) |  |  |  |  |  | |
| Q2 | 0.22 | 1.25 | 0.94 | 1.65 | 0.12 | |
| Q3 | 0.12 | 1.13 | 0.85 | 1.51 | 0.41 | |
| Q4 | 0.24 | 1.27 | 0.90 | 1.80 | 0.17 | |
| **Rainfall (per 50 mm)** | **0.01** | **1.29** | **1.12** | **1.49** | **0.001** | |
| Temperature (°C) (ref. Q1) |  |  |  |  |  | |
| Q2 | -0.12 | 0.88 | 0.66 | 1.18 | 0.40 | |
| Q3 | 0.10 | 1.11 | 0.77 | 1.61 | 0.59 | |
| Q4 | 0.42 | 1.52 | 1.00 | 2.31 | 0.05 | |
| Due to rounding, some statistically significant estimates appear to be statistically insignificant.  *Bold text indicates a statistically significant association with high anti-SGE IgG.  ^†^Rapid diagnostic test | | | | | |  |

**Table S4.** Log likelihood ratios of statistically significant *An. albimanus* high anti-SGE IgG clusters in northern and southern Haiti, 2016.

| **(Latitude, Longitude) / Radius (km)** | **Observed*** | **Expected^†^** | **LLR^‡^** | ***p*-value** |
| --- | --- | --- | --- | --- |
| **Northern Haiti^§^** |  |  |  |  |
| (19.264, -72.521) / 22.1 | 342 | 195 | 110.65 | <0.0001 |
| (19.796, -72.378) / 7.5 | 398 | 282 | 47.25 | <0.0001 |
| (19.404, -72.177) / 5.6 | 202 | 144 | 22.43 | <0.0001 |
| (19.661, -72.711) / 5.9 | 298 | 238 | 14.54 | <0.0001 |
| (20.006, -72.665) / 5.1 | 123 | 87 | 13.95 | 0.0002 |
| (19.585, -72.174) / 0.1 | 17 | 8 | 10.32 | 0.005 |
| **Southern Haiti^¶^** |  |  |  |  |
| (18.196, -73.757) / 18.5 | 885 | 713 | 76.88 | <0.0001 |

*Observed number of students with high anti-SGE IgG

^†^Expected number of students with high anti-SGE IgG

^‡^Log likelihood ratio

^§^Artibonite and Nord departments and La Tortue

^¶^Grand’Anse and Sud departments

# Supplementary Figures

**
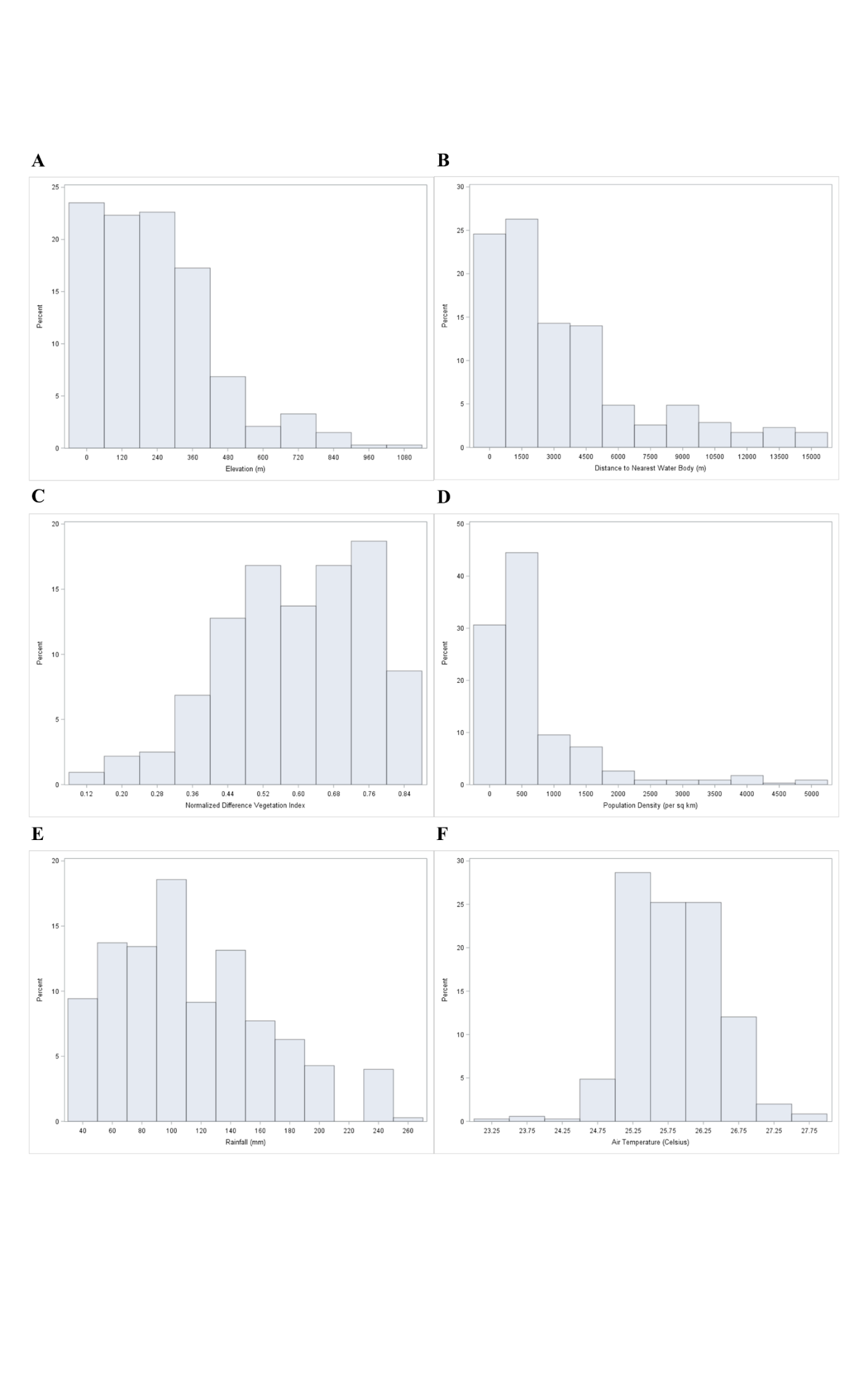
**

**Figure S1.** **Distribution of environmental covariates.** Bars depict the percentage of schools (n = 350) in northern and southern regions of Haiti that fall within ranges of values for **(A)** elevation (m), **(B)** distance to the nearest water body (m), **(C)** normalized difference vegetation index, **(D)** population density (per km^2^), **(E)** rainfall (mm), and **(F)** temperature (°C).

**
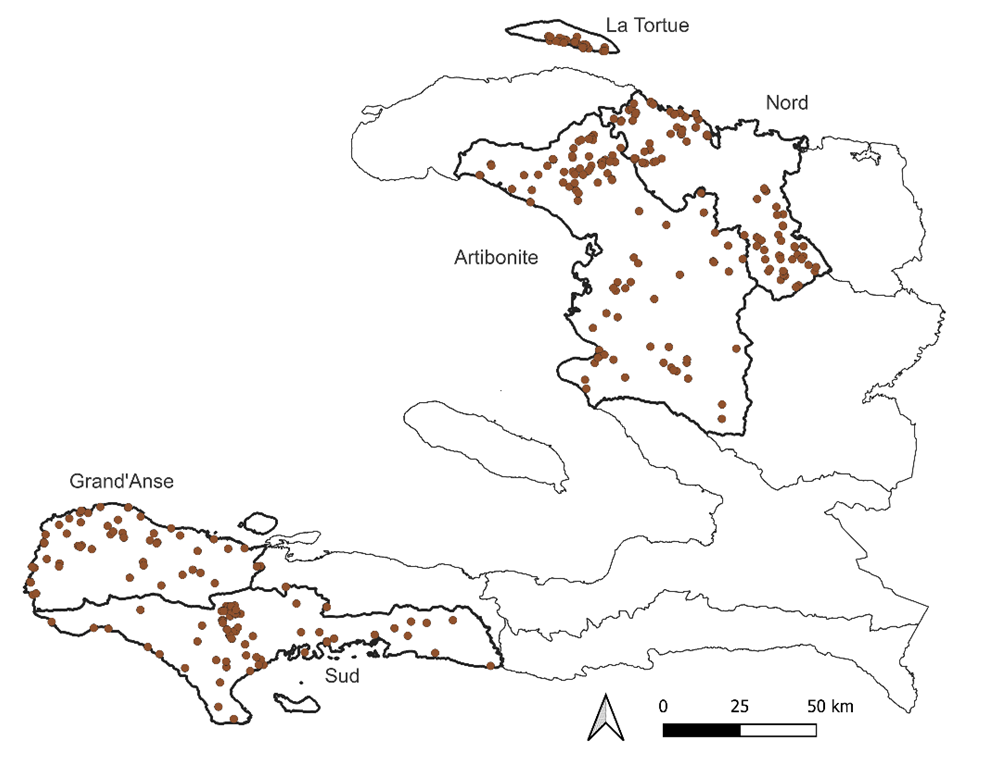
**

**Figure S2. Study enrollment sites for schools in Haiti (n = 350), 2016.**


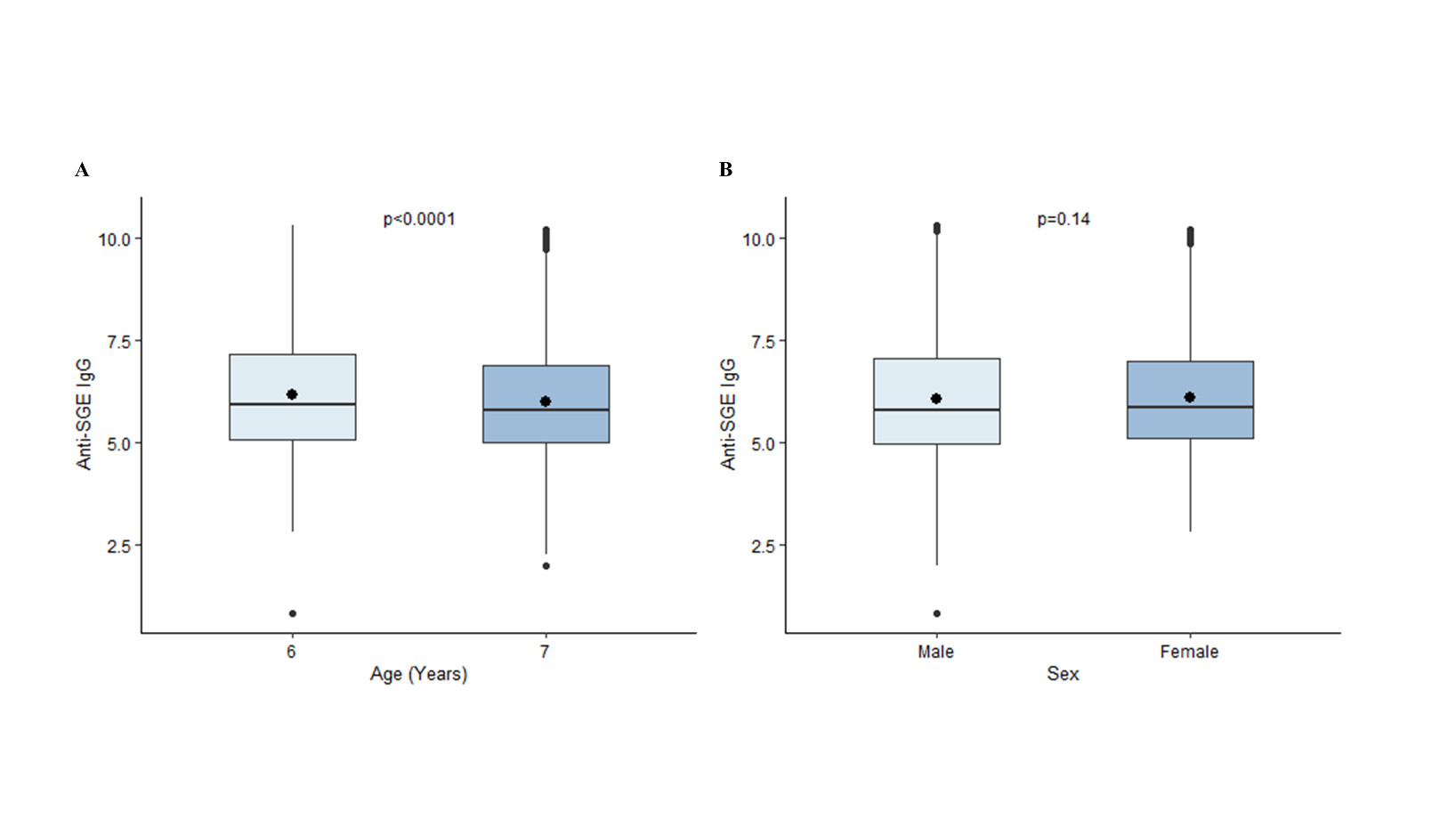


**Figure S3.** **Relationship between select individual factors and anti-SGE IgG levels. (A)** Boxplots of log-transformed salivary gland extract (SGE) immunoglobulin (Ig)G levels by **(A)** age and **(B)** sex. Boxes represent the interquartile range (IQR) of anti-SGE IgG values for each category; the horizontal line in each box is the median anti-SGE IgG value and the circle represents the mean anti-SGE IgG. Whiskers extend 1.5x IQR above and below boxes, and circles represent outlier anti-SGE IgG values outside of 1.5x IQR.


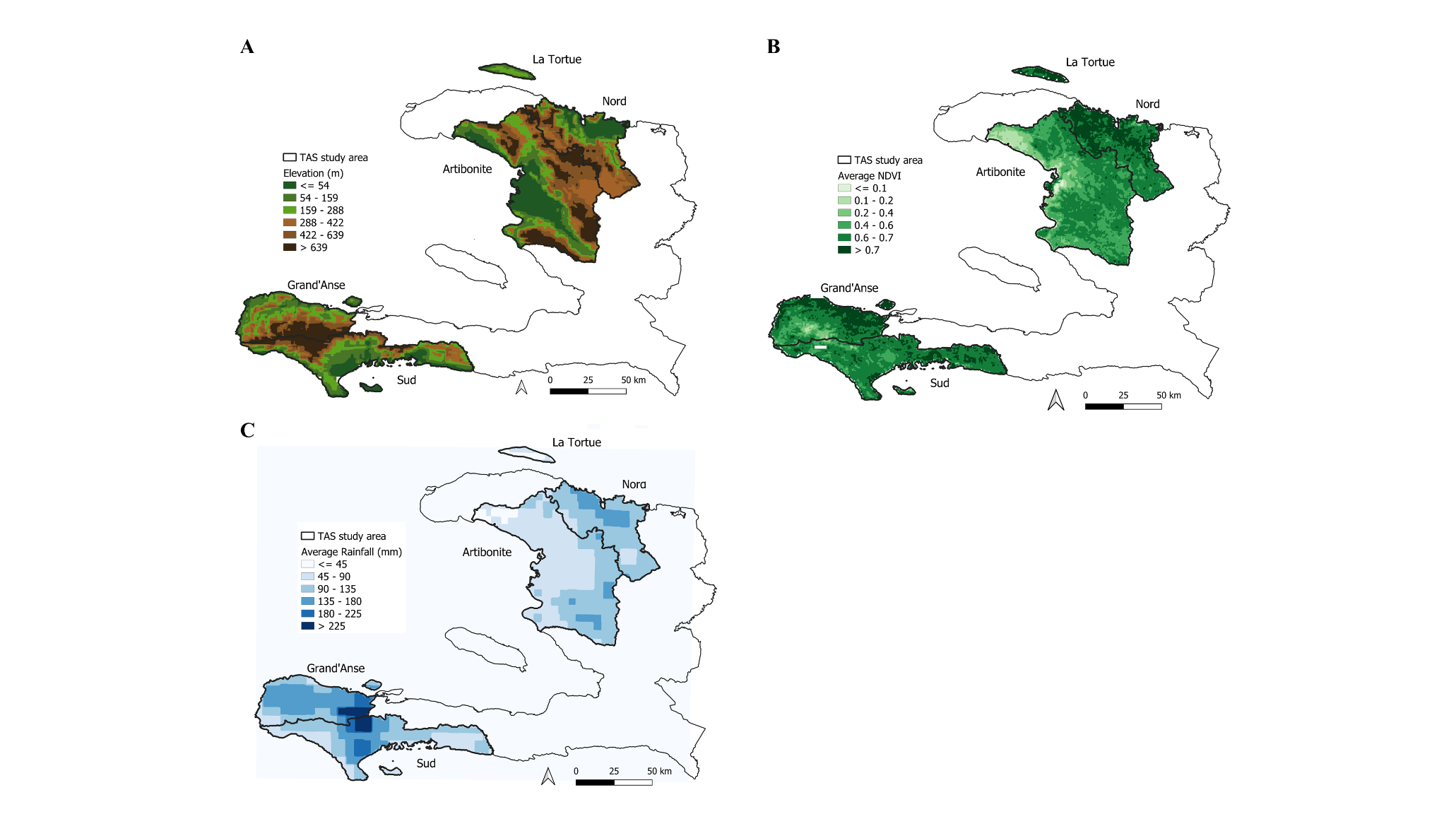


**Figure S4. Spatial distribution of select environmental variables in Haiti in 2016.** Maps of **(A)** elevation (m), **(B)** average normalized difference vegetation index (NDVI), and **(C)** average rainfall (mm) across the TAS study area.
